# Maslinic Acid Enhances Signals for the Recruitment of Macrophages and Their Differentiation to M1 State

**DOI:** 10.1155/2015/654721

**Published:** 2015-03-04

**Authors:** Cristina Sánchez-Quesada, Alicia López-Biedma, José J. Gaforio

**Affiliations:** ^1^Immunology Division, Department of Health Sciences, Faculty of Experimental Sciences, University of Jaen, Campus las Lagunillas, s/n, 23071 Jaén, Spain; ^2^Centro de Estudios Avanzados en Olivar y Aceites de Oliva, Parque Científico-Tecnológico Geolit, c/ Sierra Morena, Edificio CTSA Módulo 1, Mengíbar, 23620 Jaén, Spain; ^3^Agrifood Campus of International Excellence (ceiA3), University of Jaén, Campus Las Lagunillas, s/n, 23071 Jaén, Spain

## Abstract

The inflammatory process is involved in the genesis and evolution of different diseases like obesity, cardiovascular disease, and cancer. Macrophages play a central role in inflammation. In addition, they can regulate some stages of cancer development. Macrophages can polarize into M1 or M2 functional phenotype depending on the cytokines present in the tissue microenvironment. On the other hand, triterpenes found in virgin olive oil are described to present different properties, such as antitumoral and anti-inflammatory activity. The present study was designed to elucidate if the four major triterpenes found in virgin olive oil (oleanolic acid, maslinic acid, uvaol, and erythrodiol) are able to enhance M1 macrophage response which represents an important defense mechanism against cancer. Our results indicated that maslinic acid modulated the inflammatory response by enhancing the production of IL-8, IL-1*α*, and IL-1*β*; it promoted M1 response through the synthesis of IFN-*γ*; and finally it did not modify significantly the levels of NF*κβ* or NO. Overall, our results showed that maslinic acid could prevent chronic inflammation, which represents a crucial step in the development of some cancers.

## 1. Introduction

It is well known that patients with chronic inflammation are at a much higher risk of developing cancer. In 1863, Virchow hypothesized a link between inflammation and cancer based on the presence of leukocytes in neoplastic tissue [1]. In fact, the innate immune system, as a first line of defense, mediates the process of inflammation.* In vitro* and* in vivo* studies showed signals of inflammation in multiple pathways related to cancer development [2]. Since the evidences showed that immune cells are able to regulate almost every stage of cancer development, it would be interesting to explore biological mechanisms that could have the potential to modulate the immune response in order to reduce risks.

Macrophages play a central role in the development and maintenance of the inflammatory response. Furthermore, macrophages represent the predominant cellular type of the innate immune response found within tumors and are known as tumor-associated macrophages (TAMs). For example, breast cancer is characterized by having a large population of TAMs. Additionally, TAMs release factors to decrease the local proinflammatory antitumor response, suppressing it and providing a means of escape of the tumor cells [3].

These cells are able to synthesize a wide variety of molecules such as proinflammatory cytokines, anti-inflammatory cytokines, or proteins related with the whole inflammation process such as nuclear factor kappa beta (NF-*κβ*), which in turn can trigger the synthesis of the proinflammatory cytokine IL-1*β*. The activation of NF-*κβ* into inflammatory response could be induced by other proinflammatory molecules like nitric oxide or by feedback of proinflammatory cytokines (IL-1, TNF-*α*) [4]. TAMs come from monocytic precursors and undergo specific differentiation depending on local cues in the tissue. Macrophages can be differentiated to M1 macrophages or M2 macrophages. M1 macrophages activate type 1 helper T cells (Th1), they are able to kill pathogens, and they are tumoricidal. On the other hand, M2 macrophages activate type 2 helper T cells (Th2), they are involved in wound healing where they downregulate the inflammatory reactions and promote angiogenesis, and they have a weak tumoricidal capability [5]. TAMs are often abundantly present in malignant tumors and share many common features with the alternative activated anti-inflammatory macrophages (M2). Furthermore, these cells have been shown to enhance tumor progression by promoting tumor invasion, migration, and angiogenesis. It is well established that, depending on the stage of tumor and the kind of macrophage population present, the tumor growth could be affected. As a matter of fact, in solid tumor a high M1/M2 ratio is associated with an improved survival [6]. Thus, it seems clear that a predominance of M1 macrophage response is beneficial to modulate the inflammatory response in carcinogenesis; it could act against cancer by promoting a Th1 cytotoxic response.

**Figure 1:**
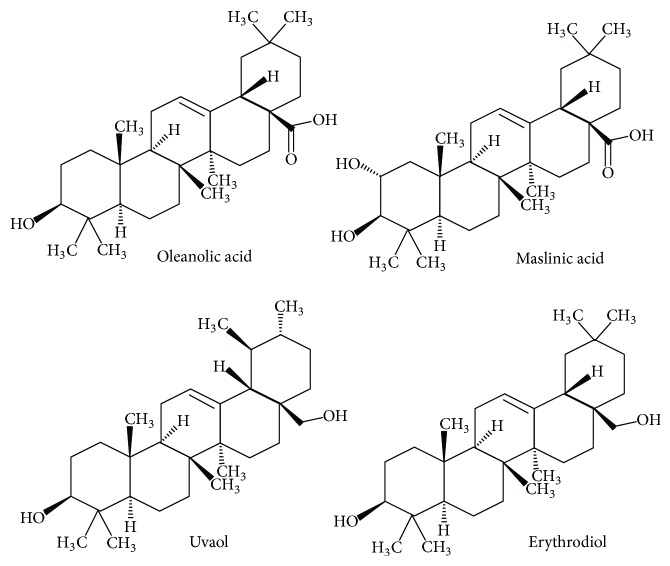
Chemical structure of oleanolic acid, maslinic acid, uvaol, and erythrodiol triterpenes.

On the other hand, oleanolic acid (OA), maslinic acid (MAS), uvaol (UV), and erythrodiol (ER) are the main pentacyclic triterpenes (Figure 1) found in both olive fruit and virgin olive oil [7], the principal source of fat on Mediterranean diet [8]. The benefits of the Mediterranean diet are well known [9, 10]. It is believed that virgin olive oil is responsible for these beneficial effects, among other things, because of its anticarcinogenic properties and protection against DNA damages [11–15]. The main triterpenes of virgin olive oil have been described to possess cardioprotective activities [16, 17], antitumor properties [7, 15], and anti-inflammatory activity [18–22]. These triterpenes are synthesized in the leaves and drupe of olive tree, and they are formed from the 2,3-oxidosqualene skeleton. The oleanolic and maslinic acids derive from the oleanane structure, while uvaol derives from ursane structure [23]. The difference between maslinic acid and oleanolic acid is an additional OH group in maslinic acid structure (carbon 2) (Figure 1).

There are no reports on the effect of triterpenes on M1 macrophage response. The present study was designed to investigate the effect of triterpenes found in virgin olive oil on M1 macrophage response.

## 2. Materials and Methods

### 2.1. Chemicals

Erythrodiol (ER) CAS [545-48-2], uvaol (UV) CAS [545-46-0], and oleanolic acid (OA) CAS [508-02-1] (purity ≥97, 98,5, and 99%, resp.) were purchased from Extrasynthese (Genay, France). Maslinic acid (MAS) CAS [4373-41-5] (purity >98%) was obtained from Cayman Chemical (Ann Arbor, MI, USA). The following were purchased from Sigma-Aldrich Co. (St Louis, MO, USA): HEPES solution; sodium pyruvate solution; nonessential amino acids mixture 100× (NEAA); lipopolysaccharides from* E*.* coli* 055:B5 (LPS); 2,3-Bis(2-methoxy-4-nitro-5-sulfophenyl)-2H-tetrazolium-5-carboxanilide inner salt (XTT sodium salt) (purity ≥90%); N-methylphenazinium methyl sulfate (PMS) (purity ≥98%); phorbol 12-myristate 13-acetate (PMA) (purity ≥99%); phosphate buffer saline (PBS); sodium chloride (NaCl) (purity ≥99,5%); L-arginine (L-Arg) (purity 98.5–101.0%) suitable for cell culture and Triton X-100. Fetal bovine serum (FBS) was obtained from PAA Laboratories GmbH (Pasching, Austria). Minimum essential medium with Eagle's salts (MEM) and phenol-red-free Roswell Park Memorial Institute 1640 medium (RPMI) were obtained from Gibco Life Technologies Ltd. (Paisley, UK). Methanol dry (maximum 0,005%), magnesium chloride (50% MgCl_2_ powder QP) (MgCl_2_), and ethanol absolute were purchased from Panreac Quimica S.L.U. (Barcelona, SPAIN). TrypLE Express was obtained from Invitrogen (Eugene, OR, USA). *β*-Mercaptoethanol was purchased from Applichem GmbH (Darmstadt, GERMANY). PIPES (98,5+%) was obtained from Acrōs Organics (Geel, Belgium). Culture plates were obtained from Starlab (Hamburg, Germany). NF*κβ* p65 (F-6) antibody was purchased from Santa Cruz Biotechnology (Santa Cruz, CA, USA). RayBio Human Cytokine Antibody Array (Human Inflammation Array I) was purchased from RayBiotech Inc. (Norcross, GA, USA).

### 2.2. Cell Line and Culture Conditions

The THP-1 (human acute monocytic leukemia) cell line was obtained from American Type Culture Collection (ATCC, Rockville, MD, USA). Cells were maintained at 37°C in a humidified atmosphere with 5% CO_2_ in MEM supplemented with 10% FBS, 1% HEPES buffer, 1% sodium pyruvate, 1% NEAA, and 0,05 mM 2-mercaptoethanol. THP-1 cells were subcultured at least twice per week, discarded, and replaced by frozen stocks after 25 passages for achieving an optimal condition of growth.

Macrophages differentiation was induced by treating THP-1 cells (1 × 10^6^ cells/mL) for 24 h with 50 nM of PMA followed by a period of further culture without PMA. PMA-differentiated THP-1 cells (1,5 × 10^5^ cells/mL) were stimulated for 24 h with LPS (1 *μ*g/mL) to acquire the M1 phenotype macrophage, and it was followed by oleanolic acid (OA), maslinic acid (MAS), uvaol (UV), or erythrodiol (ER) treatment at 1, 10, and 100 *μ*M for 4 h. All the assays were conducted under these conditions except for those specified below.

### 2.3. Cytotoxicity Assay

THP-1 cells survival, measured as the cellular growth of treated cells versus untreated cells, was carried out using an XTT-based assay according to Warleta et al. [13]. Briefly, cells were seeded into 96-well culture plates in a total volume of 100 *μ*L per well. After overnight incubation to allow cell attachment, fresh medium was added with triterpenes in a range of concentrations from 0,001 *μ*M to 100 *μ*M of OA, MAS, UV, or ER for 24 h. Thereafter, cells were incubated with XTT in phenol-red-free RPMI medium for 3 h, and absorbance was measured at 450 nm wavelength (620 nm as reference) in a plate reader (TECAN GENios Plus). Viability was calculated using the following formula:

(1) $$\begin{array}{c} \%\;\text{viable}\;\text{cells}=\left[\frac{\left(A\;\text{treated}\;\text{cells}\right)}{\left(A\;\text{control}\right)}\right]\times 100, \end{array}$$

where *A* is the difference in absorbance between optical density units (*A* = OD_450_ − OD_620_). All measurements were performed in quadruplicate and each experiment was repeated at least three times.

### 2.4. RayBio Human Cytokine Antibody Array in M1 State THP-1 Macrophages

Differentiated THP-1 cells were stimulated with LPS (1 *μ*g/mL) for 24 h. After that, cells were treated with triterpenes. A negative control (cells undifferentiated and untreated) was also tested. Then, supernatants were isolated and processed according to manufacturer instructions. Arrays membranes were directly detected using a chemiluminescence imaging system (FluorChem E System, ProteinSimple) to achieve production levels of the following cytokines/proteins: eotaxins, eotaxin-2, interleukin-1 alfa (IL-1 *α*), interleukin-1 beta (IL-1*β*), interleukin-2 (IL-2), interleukin-3 (IL-3), interleukin-4 (IL-4), interleukin-6 (IL-6), interleukin-7 (IL-7), interleukin-8 (IL-8), interleukin-10 (IL-10), interleukin-11 (IL-11), interleukin-12 p40 (IL-12 p40), interleukin-12 p70 (IL-12p70), interleukin-13 (IL-13), interferon-gamma (IFN-gamma), granulocyte colony-stimulating factor (GCSF), granulocyte macrophage colony-stimulating factor (GMCSF), chemokine CCL-1 (I-309), and metallopeptidase inhibitor 2 (TIMP-2).

Data were analyzed with the RayBio Human Inflammation Antibody Array 1 Analysis Tool (Catalogue number SO2-AAH-INF-1). Data are expressed as the relative intensity (RI) between the sample and the LPS stimulated control [RI = (AU_sample_/AU_control_)], where AU is the chemiluminescence arbitrary units acquired by the chemiluminescence imaging system.

The results are showed like the fold change (ratio of the sample value respect to the control, which was set as 1).

### 2.5. Flow Cytometry for NF*κβ* Detection in M1 State THP-1 Macrophages

After stimulation of differentiated THP-1 cells with LPS (1 *μ*g/mL), cells were treated with OA, MAS, UV, and ER at 1, 10, and 100 *μ*M. Cells were harvested with TrypLE Express and centrifugated at 300 ×g at 4°C for 10 min. The supernatant was discarded and 150 *μ*L of methanol was added. Cells were incubated 10 min at −20°C and washed with PBS. Then, 1 mL of PIPES buffer (PIPES 10 mM, NaCl 0,1 M, MgCl_2_ 2 mM, and 0,1% Triton X100 on PBS) was added to each tube. Cells were incubated at room temperature (RT) for 10 min. After that, cells were washed and suspended in anti-NF*κβ* antibody buffer (1 *μ*g/100 *μ*L) on darkness at RT for 30 min. Later, cells were washed and analyzed by flow cytometry (EPICS XL-MCL, Beckman Coulter, Spain). NF-*κβ* production was calculated using the FlowJo program (v5.7.2). Each experiment was repeated at least three independent times. Data are represented as percentage of production of NF*κβ* with respect to control, which was set as 100%.

### 2.6. NO Production in M1 Type THP-1 Macrophages

Nitric oxide (NO) production was measured according to F. Amano with some modifications [24]. Differentiated THP-1 cells (5 × 10^5^ cells/mL) were seeded on a 12-well plate and treated with OA, MAS, UV, or ER at 0.1, 1, and 10 *μ*M for 3 h. Then, LPS (1 *μ*g/mL) and L-arginine (L-Arg) at 10 mM were added to cells and incubated for 24 h. Supernatants were collected and incubated with ethanol absolute 30 min at −20°C. Supernatants were centrifuged at 10.000 ×g at 4°C for 10 min and they were aliquoted. Production of NO was measured by a NO analyzer (NOA 280i de SIEVERS, GE Water and Process Technologies, Pennsylvania, USA). Data are expressed as the percentage of NO detection relative to untreated control, which was set as 100%.

### 2.7. Statistical Analysis

For all the assays except for cytokine antibody array, data are displayed as the mean of at least three independent experiments (±SEM); for cytotoxicity assay, results are expressed as a percentage relative to the untreated control cells (which was defined as 100%). A general variance analysis (ANOVA) was carried out on data followed by Fisher's LSD test. A *P* value <0.05 was considered to be statistically significant. These statistical analyses were performed using Statgraphics Centurion XVI statistical software (Statpoint Technologies Inc., Warrenton, VA).

## 3. Results

### 3.1. Cytotoxicity Effects

**Figure 2:**
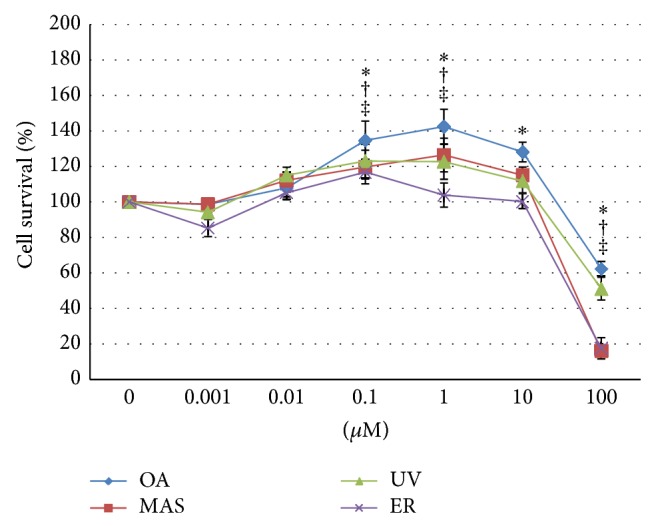
Effects of triterpenes on THP-1 macrophages survival. Cells were differentiated and treated with 0.001-0.01-0.1-1-10-100 *μ*M of oleanolic acid (OA^*^), maslinic acid (MAS^†^), uvaol (UV^‡^), and erythrodiol (ER^Δ^) at 24 h. ^(^^*^^)(†)(‡)(Δ)^Statistically significant differences compared with cells untreated (*P* < 0.05).

Cell survival was determined by the XTT assay. THP-1 cells were differentiated and exposed to increasing concentrations (from 0,001 *μ*M to 100 *μ*M) of OA, MAS, UV, and ER for 24 h. Our results showed that the four triterpenes assayed decreased significantly cell viability at 100 *μ*M, whereas, at low concentrations, they did not show cytotoxic effects (Figure 2).

### 3.2. RayBio Human Cytokine Antibody Array

**Figure 3:**
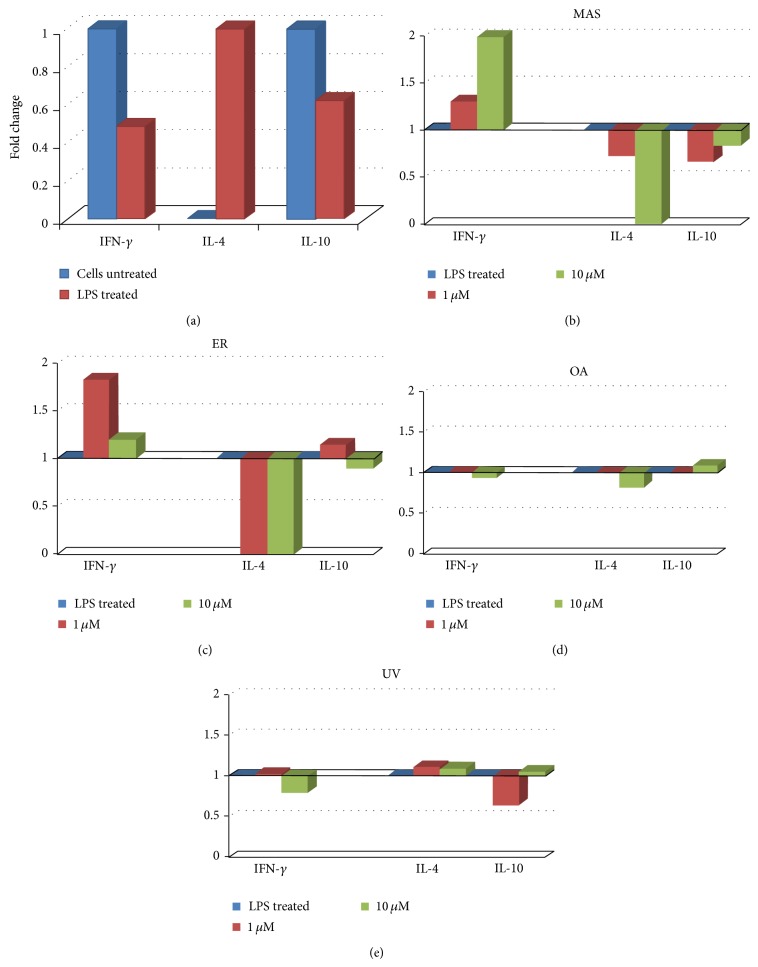
M1 polarization related cytokines production versus M2 polarization related cytokines production in cell unstimulated and stimulated with LPS (a) and in M1 polarized THP-1 macrophages after treatment with MAS (b), ER (c), OA (d), and UV (e) at 1, 10, and 100 *μ*M. Results are expressed as the fold change in RI (relative intensity) related to stimulated control which was set as 1.

**Figure 4:**
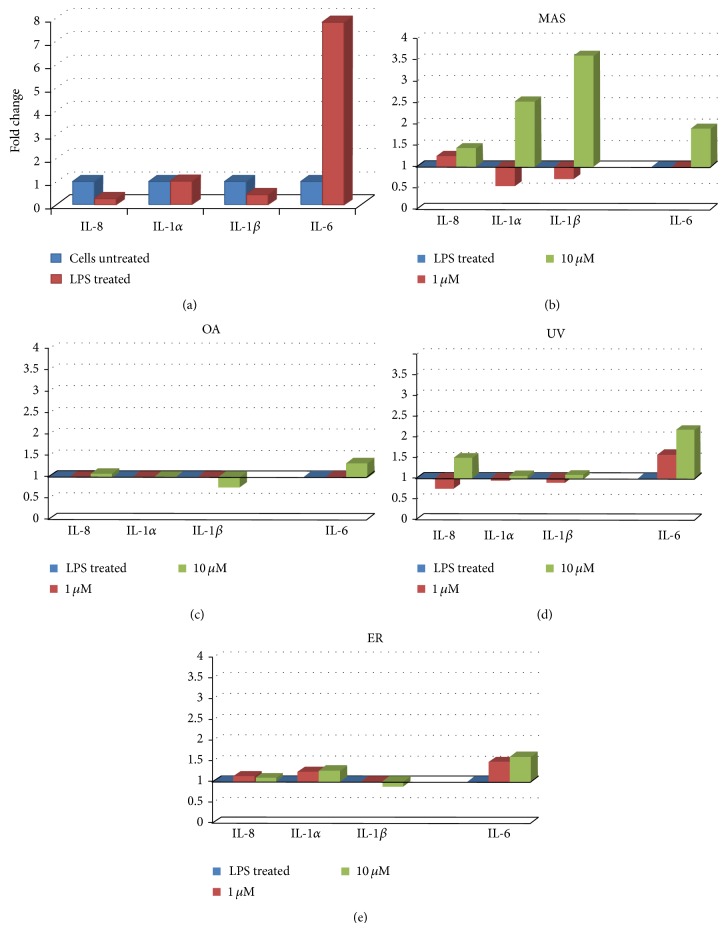
Production of cytokines responsible of macrophage recruitment produced in cell unstimulated and stimulated with LPS (a) and in M1 polarized THP-1 macrophages treated with MAS (b), OA (c), UV (d), and ER (e) at 1, 10, and 100 *μ*M.

Production of inflammation-related proteins was measured on THP-1 macrophages cells stimulated with LPS (1 *μ*g/mL) for differentiation of M1 phenotype. All the inflammation-related proteins showed significant differences in LPS stimulated cells with respect to untreated cells (Figures 3(a) and 4(a)).

#### 3.2.1. M1/M2 Polarization Related Cytokines

After triterpenic treatments we observed that IFN-*γ* level, which leads to M1 polarization, was increased with respect to control at MAS 1 *μ*M, 10 *μ*M and ER 1 *μ*M (Figures 3(b) and 3(c)). For the rest of compounds, IFN-*γ* production levels were similar to control (Figures 3(d) and 3(e)). However, IL-4, which leads to M2 polarization, decreased levels after MAS 1 *μ*M treatment and is absent after MAS 10 *μ*M, ER 1 *μ*M, and 10 *μ*M treatment (Figures 3(b) and 3(c)). IL-10 did not show any significant differences with respect to control. In the other triterpenes tested there were not differences with respect to control (Figures 3(d) and 3(e)). At the concentration of 100 *μ*M, most of the compounds have strong differences with respect to the control, but it might be due to the cytotoxic effects that they exerted at elevated concentrations.

#### 3.2.2. Macrophages Recruitment-Related Cytokines and Proinflammatory Cytokines

Cytokines related with macrophages recruitment such as IL-8, IL-1 alpha, and IL-1 beta appeared increased in macrophages after treatment of MAS at 10 *μ*M. The production of IL-6 cytokine increased at the same concentration (Figure 4(b)). For the rest of compounds only the IL-8 cytokine production was increased at UV 10 *μ*M and IL-1 alpha at ER 10 *μ*M. The IL-6 cytokine levels were increased in all the treatments at 10 *μ*M and at ER 1 *μ*M (Figures 4(c), 4(d), and 4(e)).

For the rest of cytokines and proteins related with inflammation, the signals were closed to background (data not shown).

### 3.3. Effects on NF-*κβ* Production

Detection of NF-*κβ* (p65) was performed by flow cytometry in differentiated THP-1 cells stimulated with LPS 24 h and treated with 1, 10, and 100 *μ*M of OA, MAS, UV, or ER triterpenes. There were not statistically significant differences between control and samples (Table 1).

### 3.4. NO Production

**Figure 5:**
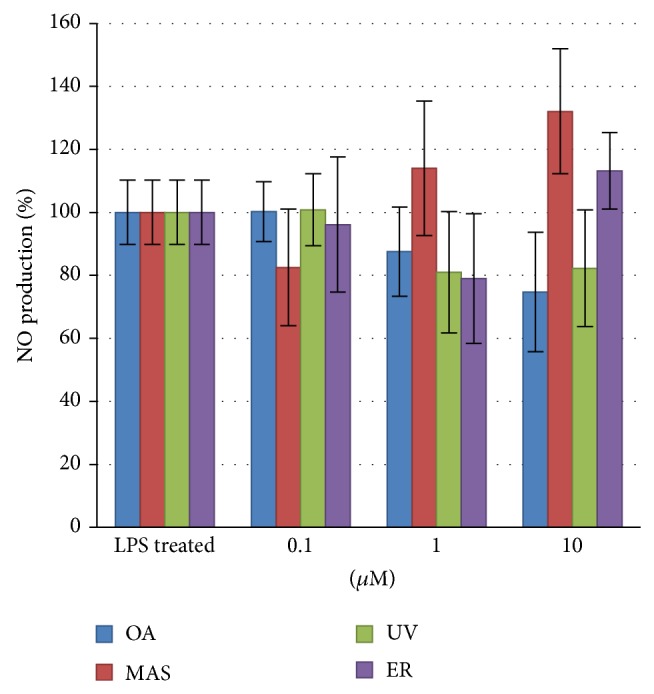
NO production of M1 polarized THP-1 macrophages treated with 0.1, 1, and 10 *μ*M of OA, MAS, UV, and ER. Data are expressed relative to LPS treated cells, which was established as 100%. Not statistical differences were found.

NO production was measured on M1 phenotype THP-1 macrophages at 0.1, 1, and 10 *μ*M of OA, MAS, UV, or ER. Although any treatment exhibited a statistically significant variation compared with the LPS stimulated control, a slight increase of NO production was observed at MAS 1 *μ*M, 10 *μ*M, and ER 10 *μ*M and a decrease at OA 10 *μ*M and ER 1 *μ*M (Figure 5). LPS stimulated control showed statistical differences with respect to unstimulated control (data not shown).

## 4. Discussion

The THP-1 cell line has a closed gene expression to primary macrophages, derived from peripheral blood mononuclear cells, in contrast to other monocytes cell lines like U937 [25]. Furthermore, a PMA differentiation of THP-1 cells drives cells to a differentiated macrophage phenotype that seems very nearby to monocyte-derived human macrophages [26]. Analysis of primary macrophages in culture will always provide more truthful information about inflammation response than cellular models, but these primary cultures are also difficult to culture in the quantities required to allow biochemical analysis. Thus, PMA differentiated and LPS stimulated THP-1 cells represent a useful experimental model to study the inflammatory response and their modulation after treatment with food compounds [27]. Moreover, recently the consequent polarization to M1 phenotype that LPS promotes in THP-1 macrophages has been described [28]. Thus, THP-1 macrophages are the best option for study* in vitro* effects of certain compounds in macrophages differentiated to M1 phenotype.

According to this, THP-1 cells were used to study* in vitro* effects of OA, MA, UV, and ER on M1 macrophages. THP-1 macrophages were differentiated into the M1 stage by LPS treatment.

Macrophages constitute an extremely heterogeneous population, which polarize into distinct macrophages types, mainly identified as M1 (or classically activated) and M2 (or alternatively activated) [29]. Previously, our group described two phenotypically and functionally different populations among splenic macrophages in response to* C. albicans* infection. One of them (M2 phenotype) expressed high levels of major histocompatibility complex (MHC) class II surface expression and is poorly phagocytic. The other one (M1 phenotype) expressed low levels of MHC class II molecules and is highly phagocytic [30]. We suggested that NK cells prime splenic macrophages were phagocytic in naïve BALB/c mice, probably mediated by IFN-*γ* production, the same signal that monocytes need in tumor microenvironment for polarized to M1 phenotype. Thus, infections as well as cancer could polarize macrophages to M1 or M2 phenotypes depending on the microenvironment signals [6].

In nonprogressing or regressing tumors, TAMs are related to a classic macrophage activation M1-like program, characterized by proinflammatory activity, antigen presentation, and tumor lysis. Even more, a high M1/M2 polarization ratio improved survival in lung carcinoma [6]. In malignant tumors, TAMs resemble M2 phenotype. These macrophages increase angiogenesis, tumor cell extravasation, and growth; they suppress activation of dendritic cells, cytotoxic T lymphocytes, and natural killers [31, 32].

M1 macrophages appear to have a proangiogenic function early in tumorigenesis [33], when the tumor needs blood vessels formation to grow; this fact supports the idea of the role that M1 plays in the early stages of breast tumor formation and it seems to be one of the first immune cells present in the inflammatory process. But, in advanced breast cancers, macrophages resemble the M2 phenotype, while M1 phenotype has not been found; this is the reason why TAMs are generally related more to a M2 phenotype than M1. Further, M2 macrophages express changes in several metabolic pathways, controlling the inflammatory response by downregulating M1-mediated functions. It seems that tumor cells are able to produce several signals that polarize monocytes to M2. This preferential polarization is the result of absence of M1-orienting signals, such as INF-*γ* or bacterial components in the tumor [29].

We hypothesized that, in established solid tumors, the activation of a M1 response could be a useful strategy in order to prevent tumor growth.

**Figure 6:**
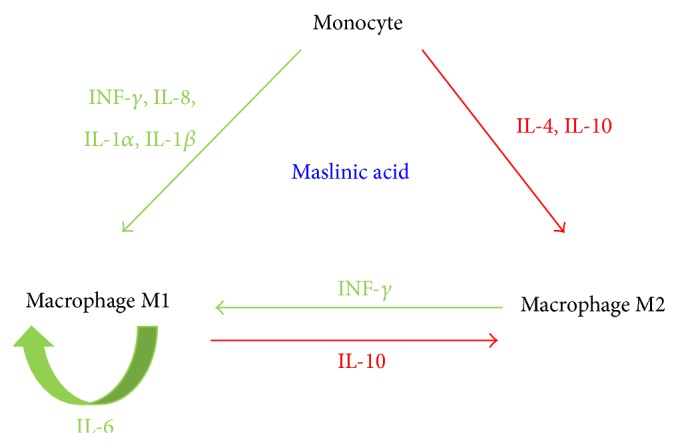
Role of maslinic acid in differentiation and recruitment of macrophages. Cytokine produced after MA treatment in M1 macrophages is represented in green and cytokine inhibited after MA treatment in M1 macrophages is represented in red.

Our results show that MAS and ER at low concentration increased the production of INF-*γ* in M1 polarized THP-1 macrophages. By this way, M1 macrophages could mediate and control their own response differentiating monocytes to M1 instead of M2 in carcinogenesis. This increase of INF-*γ* production would be a proinflammatory signal for monocytes in inflammation locations, differentiating these monocytes to M1 phenotype (Figure 6), making more efficient the recognition of neoplastic cells, and mediating an effective Th1 cells response.

On the other hand, M2 macrophages can switch to M1 at the site of the tumor by INF-*γ* induction and receptor-mediated activation signals to promote tumor regression [6]. With MAS and ER, it could be M1 polarized macrophages theirselves, which could hypothetically reprogram the M2 macrophages that could show up at the sites of tumor formation, to M1 state.

Indeed, MAS at 10 *μ*M and UV at 10 *μ*M showed levels of IL-8 production slightly higher than the control (Figures 4(b) and 4(d)). Although IL-8 production was not statistically significant, IL-1*α* and IL-1*β* were dramatically increased. It is known that the role of IL-8 cytokine on the monocyte recruitment as well as CXCL12 chemokine, whose precursors are IL-1*α* and IL-1*β*, interestingly increased in MAS at 10 *μ*M (Figure 4(b)).

Apart from fortifying the proinflammatory response by activating monocytes to M1 and preventing the M2 polarization of monocytes at sites of inflammation, MA appears to promote the recruitment of more cells that could support the immune response on the inflammation location.

Furthermore, some authors describe that NF-*κβ* promotes the presence of immunosuppressive M2 phenotype [34]. In order to assess the possible role of triterpenes in promoting production of NF-*κβ*, we studied its production by the M1 phenotype THP-1 macrophages. It is important to note that a high increase of NF-*κβ* expression may lead to an aggravated inflammatory response that could guide to a consequent chronic inflammation [34]. In our study, levels of NF-*κβ* were not statically significant with respect to control untreated, so these M1 macrophages treated with triterpenes did not show to promote chronic inflammation.

Furthermore, nitric oxide (NO) production by NOS (nitric oxide synthase) supports this point. At normal levels, NOS acts like protector against injury, but at elevated levels in the tissue it has been described like an inflammatory enzyme that promotes carcinogenesis [35]. We studied the levels of NO after treatment with triterpenes and there was no statistically significant change in their production compared to the control. These results agree with those of Márquez-Martín et al., who described an inhibition of NO production in peritoneal murine macrophages upon exposure to MA treatment [36]. At 0.1 *μ*M we notify the reduction of NO after MA treatment in M1 macrophages.

It is important to note that, at the highest concentration, these triterpenes are cytotoxic for THP-1 macrophages but the effects of these triterpenes in M1 macrophages focus at low concentrations.

## 5. Conclusion

Maslinic acid possesses two principal actions on M1 macrophages: first, it enhanced recruitment of macrophages by production of IL-8, IL-1*α*, and IL-1*β*; and second, it promoted M1 response through the synthesis of IFN-*γ*.

Further studies are needed for assessing the action of these macrophages treated with triterpenes in carcinogenesis. However, maslinic acid could be a useful natural compound to modulate inflammation response.

## Figures and Tables

**Table 1:** NF*κβ* production by M1 polarized THP-1 macrophage cells treated with OA, MAS, UV, and ER at 1, 10, and 100 *µ*M along 4 h, measured by flow cytometry. Data are expressed like the percentage of NF*κβ* production with respect to cells LPS treated, which was set as 100%. Standard error means (SEM) represented as percentage. Not statistical differences found at *P* < 0.05.

Treatment	Concentration	Mean	SEM
LPS treated		100	±66,05

OA	1 *µ*M	132	±12,06
	10 *µ*M	74	±17,15
	100 *µ*M	84	±15,74

MAS	1 *µ*M	96	±13,26
	10 *µ*M	101	±17,16
	100 *µ*M	116	±30,50

UV	1 *µ*M	112	±19,54
	10 *µ*M	121	±29,72
	100 *µ*M	102	±23,86

ER	1 *µ*M	110	±38,91
	10 *µ*M	101	±28,62
	100 *µ*M	99	±23,88
